# Think Twice before Prescribing Antibiotics for That Swollen Knee: The Influence of Antibiotics on the Diagnosis of Periprosthetic Joint Infection

**DOI:** 10.3390/antibiotics10020114

**Published:** 2021-01-26

**Authors:** Graham S. Goh, Javad Parvizi

**Affiliations:** Rothman Institute, Thomas Jefferson University, Philadelphia, PA 19107, USA; Graham.Goh@rothmanortho.com

**Keywords:** knee arthroplasty, hip arthroplasty, antibiotics, infection, periprosthetic joint infection, diagnosis, culture, aspiration, molecular, synovial fluid

## Abstract

Periprosthetic joint infection (PJI) is a rare but devastating complication after total joint arthroplasty. An estimated 7–12% of patients have negative cultures despite clear clinical evidence of infection. One oft-cited reason for this occurrence is the administration of antibiotics in the weeks prior to obtaining cultures. This article reviews the influence of antibiotics on the diagnosis of PJI. Specifically, we examine the effect of prophylactic and therapeutic antibiotic administration on the diagnostic accuracy of microbiological cultures as well as serum and synovial biomarkers. We also explore the potential of molecular techniques in overcoming these limitations in patients who have received antibiotics before specimen collection and propose areas for future research.

## 1. Introduction

Periprosthetic joint infection (PJI) is a rare but devastating complication after total joint arthroplasty (TJA). The risk of PJI following total knee arthroplasty (TKA) varies between 0.5% and 2% [1], whereas a slightly lower risk of 1% has been observed for total hip arthroplasty (THA). Despite the low incidence of this complication, PJI is the most common indication for revision TKA in the United States [2] and the third most common indication for revision THA [3]. As the population ages and the demand for joint arthroplasty continues to grow over the next decade [4], the prevalence of PJI will invariably increase, posing a substantial economic burden to the healthcare system [5]. It is therefore imperative that clinicians obtain a timely and accurate diagnosis to avert the consequences of this disastrous complication. 

In addition to a thorough history and physical examination, the diagnosis of PJI often relies on serological tests and radiographic evaluation [6]. In particular, isolating the infecting microorganism from cultures of fluid or tissue within the joint remains the cornerstone for diagnosis and targeted antibiotic therapy, which has been shown to increase the chances of treatment success [7]. This also provides valuable prognostic information for patients and guides perioperative counseling [8]. According to the recent definition of PJI proposed by the European Bone and Joint Infection Society (EBJIS), isolation of the same pathogen from two separate intraoperative tissue or fluid samples is diagnostic of PJI [9]. However, while multiple clinical guidelines on the appropriate surgical and laboratory techniques to maximize culture yield have been published [10], an estimated 7–12% of patients still have negative cultures despite clear clinical evidence of infection, such as a draining sinus or a high synovial fluid white blood cell (WBC) count [8,11,12]. An oft-cited reason for this occurrence is the administration of antibiotics in the weeks prior to obtaining cultures [11], prompting the guidelines of major orthopedic associations to recommend against this practice until the diagnosis of PJI has been reliably established [13].

This narrative review discusses the influence of antibiotics on the diagnosis of PJI. Specifically, we examine the effects of antibiotic administration on the diagnostic accuracy of microbiological cultures as well as serum and synovial biomarkers. We also explore the potential of molecular techniques in overcoming these limitations in patients who have received antibiotics before specimen collection and propose areas for future research. 

## 2. Premature Antimicrobial Administration in Hematogenous PJI

Hematogenous PJI, a subtype of PJI, remains a unique clinical entity as patients suffering from this complication are often at a higher risk of premature antimicrobial administration to treat the source of infection, prior to implant revision or even the onset of symptoms for an infected joint. This type of PJI often manifests in the late postoperative period after an uneventful course and is usually caused by hematogenous spread from a distant infectious focus. The incidence of late PJIs (manifesting 2 years post-implantation or later) is estimated at 0.07% per prosthesis-year [14], and the proportion of hematogenous PJI is estimated at 20–35% of all PJI episodes [15]. As *Staphylocccus aureus* is the most common cause of bacteremia [16] and the majority of patients with *S. aureus* PJI obtain this infection via the hematogenous route [17], it is often assumed that most cases of hematogenous PJI are caused by *S. aureus*. However, it is important to note that this is an erroneous presumption, since recent studies have shown that *S. aureus* is only responsible for 28–41% of such cases and Streptococcal species are also highly prevalent [15,18]. With regards to the source of hematogenous PJI, a recent study by Rakow et al. showed that the cardiovascular system (including native or prosthetic valve endocarditis, implantable device-associated infections, and central or peripheral catheter-related infections) was the site of primary infection in 68% of cases [18]. In contrast, Zeller et al. determined that the skin (15%) and teeth (11%) were the most common portals of entry in hematogenous PJIs [15], although it remains uncertain whether “skin” infections also encompassed infected intravenous sites, as was done in historical studies [19]. Notwithstanding, it is important that all physicians be familiar with the epidemiology, origins, and microbiological features of hematogenous PJI, as this can guide treatment decisions in regards to whether early administration of antibiotic therapy is warranted, or whether such treatment may be premature and should be deferred till after microbiological isolation of the PJI organism is performed. These decisions have important clinical implications on the diagnosis of PJI and may do more harm than good in most cases. The influence of antibiotic administration on the accuracy of various laboratory tests used in the diagnosis of PJI is outlined in the following sections.

## 3. Antibiotics and Culture Yield

### 3.1. Therapeutic Antibiotics

The gold standard for the diagnosis of PJI has traditionally been the isolation of an organism from microbiological cultures obtained intraoperatively. However, this is not always possible despite clinical evidence confirming the presence of PJI—a phenomenon commonly referred to as culture-negative periprosthetic joint infections (CN-PJI) [11]. The prevalence of CN-PJI has been noted to be as high as 40% [12,20,21]. Possible reasons for negative cultures have been proposed, such as infection by fastidious pathogens, biofilm encapsulation, uncommon organisms (e.g., fungi or mycobacteria) that do not replicate on routine culture media, inadequate sampling or transportation, as well as insufficient resuscitation in the laboratory [10,22]. Nonetheless, the most important cause of failure to isolate an organism from intraoperative cultures is the administration of antibiotics before obtaining samples from the infected joint [11,12,23]. Sub-therapeutic or mistargeted antimicrobial treatment has been shown to induce a viable but non-culturable (VBNC) physiological state in many pathogens [24,25,26,27], rendering the results of these cultures falsely negative. This cellular state is characterized by low metabolic activity and the absence of growth on routine bacteriological media [28]. Metabolic activity and culturability can be restored if the appropriate nutritional stimulation is provided—this process is known as resuscitation [29]. Unfortunately, antimicrobials that act on growing cells are often unable to eradicate VBNC cells, likely because their metabolic activity has decreased to such an extent that they effectively become resistant to treatment [30]. For instance, vancomycin was found to be effective against VBNC *Enterococcus faecalis* cells only when administered at five hundred times the minimum inhibitory concentration [31]. While most pathogens are generally unable to cause infection when in a VBNC state, these bacteria still retain their virulence and can cause infection after being resuscitated [25]. VBNC microorganisms have been postulated to be the cause of re-infections in patients who initially experience remission following antimicrobial therapy, as in the case of recurrent gastric ulceration by *Helicobacter pylori* or recurrent urinary tract infections by *Escherichia coli*. These findings likely account for the phenomenon of CN-PJI, especially in the setting of prior antibiotic administration. False-negative results not only preclude the selection of targeted antimicrobial therapy and lead to lower rates of treatment success [32], but also result in unnecessary anxiety for patients who may challenge the diagnosis of PJI due to an inability to isolate a pathogen [12]. Furthermore, empirical treatment of CN-PJI usually entails administering broad-spectrum or multiple antibiotics to cover the most common microorganisms according to epidemiological surveys, which may be less effective and increases the risk of adverse reactions or systemic toxicity. Consequently, it is imperative that extra efforts be made to isolate the infecting organism in all cases of PJI. 

Culture-negative infections have been reported in multiple fields in clinical medicine [33,34,35,36]. A recent multicenter, single-group, diagnostic study of 325 patients with severe sepsis reported a significant difference in the proportion of positive blood cultures in patients who had specimens taken pre- (31.4%) and post-antimicrobial (19.4%) administration, indicating that the initiation of empirical therapy reduced the sensitivity of blood cultures [35]. In addition to its implications on culture yield, septic patients who have cultures after initiation of antimicrobials may also have longer intensive care unit (ICU) and hospital stays, as well as greater mortality risk [34]. Current evidence in orthopedic surgery also cautions against the use of antibiotics in the period leading up to revision arthroplasty [11,23,37,38]. Trampuz et al. demonstrated that any administration of antibiotics in the two weeks before obtaining intraarticular cultures adversely influenced the sensitivity of cultures and was associated with a higher false negative rate (55% vs. 23%) [37]. In another case-control study of 60 patients, Berbari et al. found that 53% of patients who had CN-PJI received antimicrobial therapy within three months before the diagnosis and 23% received the antimicrobial agent up to the time samples were taken from the infected joint [11]. Similarly, Malekzadeh et al. found that patients with CN-PJI were four times more likely to have received antimicrobial therapy in the preceding three months before diagnosis [23]. In the same vein, Shahi et al. reported that patients with antibiotic use before aspiration had a higher rate of CN-PJI compared to those without any antibiotic history [38]. Given these considerations, clinical practice guidelines have recommended against preemptive treatment before a thorough evaluation for PJI, advising clinicians to withhold antibiotic therapy for at least two weeks before intraoperative specimen collection to improve culture yield [13]. For the same reason, the Infectious Diseases Society of America (IDSA) clinical practice guidelines further recommend against antibiotic therapy for at least two weeks before joint aspiration to increase the chances of isolating the causative organism [39]. Based on the available literature, the general consensus is to discontinue antibiotic therapy for a minimum of two weeks prior to surgical intervention or culture. However, whether these recommendations can be applied uniformly to all suspected cases of PJI remains unknown. In particular, several authors have proposed that an even longer period without antimicrobial exposure may be required to culture certain fastidious organisms [37,40,41]. Future research is needed to refine the present guidelines with regards to the effect of different antimicrobial agents on the culture yield of differing organisms, as well as to define the optimal antibiotic-free period before obtaining samples in patients with suspected PJI.

### 3.2. Prophylactic Antibiotics

It is important to make a distinction between therapeutic antibiotics (which often requires a prolonged course of treatment) and prophylactic antibiotics (which often comprises a single dose administered perioperatively [42]). While the abovementioned studies have demonstrated that antibiotic administration prior to identifying the causative pathogen increases the risk of false-negative cultures [23], the need to withhold pre-incision prophylactic antibiotics remains a controversial issue in orthopedic surgery [43,44,45,46,47]. Prophylactic antibiotics were traditionally believed to interfere with culture yields from intraoperative samples, leading some investigators to advocate against their use in the context of revision arthroplasty for suspected PJI [40,48]. Although this practice appears logical, withholding prophylactic antibiotics may increase the risk of surgical site infection or systemic dissemination perioperatively. Moreover, recent evidence has largely refuted this belief [43,44,45,46,47]. Two randomized controlled trials also demonstrated identical rates of positive intraoperative cultures [43] and concordant cultures [44] in patients who did or did not receive prophylactic antibiotics before incision. Utilizing intraoperative controls, Bedencic et al. obtained samples from the same surgical site before and after cefazolin infusion and found no difference in the mean number of colony-forming units (CFU) per gram of tissue between the two sets of cultures. Importantly, the tissue concentrations of cefazolin were all higher than the minimum inhibitory concentration at the time of obtaining the second sample [45]. More recently, a large cohort study of 425 revision TKAs reported no difference in the number of positive cultures between non-prophylaxis and prophylaxis groups (26% vs. 27%) [47], and the species of bacteria cultured were also similar. Furthermore, as a trend toward a higher rate of PJI in the early postoperative period was found in the group who did not receive antibiotic prophylaxis (6.4% vs. 1.6%), the authors cautioned against withholding prophylactic antibiotics for patients undergoing aseptic revision arthroplasty. Given the large body of evidence suggesting that the practice of withholding prophylactic antibiotics to maximize culture yield may not be as critical as previously thought, the 2018 International Consensus Meeting (ICM) recommended that perioperative antibiotic administration for revision TJA should not be routinely withheld, but should instead be guided by the degree of clinical suspicion for PJI and whether or not a causative organism has been isolated before surgery [10].

## 4. Antibiotics and Biomarkers

Serum and synovial biomarkers are useful adjuncts in the diagnosis of PJI [6], especially in the absence of major criteria such as a communicating sinus tract or two positive cultures [49]. Biomarkers are measurable biological substances that are part of a physiological or pathological pathways or the pharmacological response to therapeutic interventions [50]. As with its impact on culture yield, antibiotic administration prior to obtaining blood or synovial fluid samples may also adversely affect the accuracy of common biomarkers used to support the diagnosis of PJI. Shahi et al. found that a higher percentage of biomarker values were below the Musculoskeletal Infection Society (MSIS)-determined threshold for erythrocyte sedimentation rate (ESR), C-reactive protein (CRP), and synovial polymorphonuclear cell percentage (PMN%) in patients who received antibiotics compared to patients who did not, indicating a higher rate of false-negatives [38], and median biomarker values were also lower in the group with antibiotic use. Two possible mechanisms may account for these findings. Firstly, antibiotics have been shown to display anti-inflammatory properties by inhibiting interleukin (IL)-1 and tumor necrosis factor (TNF) production [51], and these effects may be more pronounced in certain classes of antibiotics including macrolides and quinolones [52,53]. Secondly, antibiotics may decrease the inflammatory response indirectly by decreasing bacterial load and macrophage activation. This leads to an attenuation of the cytokine cascade, reduction in IL-1, TNF, and IL-6, and decrease in inflammatory markers such as ESR and CRP [54]. As synovial fluid cell counts and differentials fluctuate according to serum levels [55,56], this will concomitantly cause a decline in synovial biomarkers. These mechanisms support the claim that antibiotics administration in patients with suspected PJI affects the values of common serologic and synovial biomarkers used in diagnostic criteria. Future research could be directed at establishing new cutoffs for PJI diagnosis in patients who inadvertently receive antibiotics before a diagnostic workup.

Newer serum and synovial fluid biomarkers play an integral role in the diagnosis of PJI and have been incorporated into recent guidelines as minor diagnostic criteria [49]. Serum IL-6 has been established as a valuable inflammatory marker in association with sepsis, trauma, and major surgery. Given that IL-6 lies upstream of traditional biomarkers in the inflammatory cascade, it is postulated to be a more rapid and sensitive blood test for the detection of PJI [57]. Berbari et al. found that IL-6 had the highest accuracy in diagnosing PJI when compared to ESR and CRP [58]. A growing interest in the use of IL-6 has led to its incorporation into the latest clinical practice guidelines [13]. Notwithstanding, current barriers to its use include the relatively high cost and technical skills required to run the analysis. As serum IL-6 assays become more widely available for clinical use, this biomarker could be used in combination with other routine markers like CRP, further enhancing their diagnostic yield as shown in previous studies [59]. Synovial fluid biomarkers with a high specificity for PJI include leukocyte esterase and human alpha-defensin. Leukocyte esterase is an enzyme secreted by activated neutrophils following their migration to the site of infection. Its use has gained recognition in the diagnosis of urological infections, and more recently, PJI [60]. Leukocyte esterase tests are readily available, point-of-care tests requiring the application of infected joint fluid onto colorimetric strips. Detection of the enzyme is then reflected as a color change on the test strip [60]. Despite its utility, one major limitation is that the contamination of fluid samples with blood can interfere with the colorimetric changes of the test strip [61], although this may be overcome by centrifuging samples prior to application. Tischler et al. showed that leukocyte esterase testing had a high specificity and moderate sensitivity in the diagnosis of PJI [61], while Wetters et al. reported a sensitivity of 92.9–100%, and specificity of 77.0–88.8% [62]. Alpha defensin is another synovial fluid biomarker with high accuracy when used in the diagnostic workup for PJI [63]. Defensins are antimicrobial peptides that act on most Gram positive and negative bacteria, fungi, and enveloped viruses [64]. They are commonly secreted by neutrophils as well as certain macrophage cell lines, and their synthesis is induced by pro-inflammatory cytokines or microbiological products. While their precise antimicrobial mechanism has yet to be fully elucidated, alpha-defensins are generally believed to cause a disruption in pathogen membrane integrity, resulting in cell lysis [64,65]. Previous studies have demonstrated the utility of alpha-defensin as a diagnostic tool for PJI [66]. Of note, alpha-defensin provides consistent accuracy irrespective of the infecting organism species or virulence [63], with studies reporting a sensitivity and specificity of over 95% for the diagnosis of PJI [66,67,68]. Bingham et al. even suggested that the diagnostic accuracy of synovial fluid alpha-defensin assays exceeded that of other available tests [66]. These encouraging results ultimately led to its incorporation into previous diagnostic criteria for PJI [49]. 

Interestingly, the effect of preoperative antibiotic administration on the sensitivity of more recent biomarkers like alpha defensin and leukocyte esterase has been found to be negligible. Deirmengian et al. analyzed the results from a single institution and did not find any effect of pre-aspiration antibiotic administration on alpha-defensin levels or sensitivity [68]. These findings were corroborated by Shahi et al. in a multicenter study of 106 PJIs, who reported that the 30 cases treated with antibiotics before diagnostic workup had a similar median alpha-defensin level compared to the 76 untreated cases, suggesting that alpha-defensin was more sensitive than ESR, CRP, fluid PMN%, and fluid culture when screening for PJI in the setting of antibiotic use [69]. In another study, the same authors found that the administration of antibiotics resulted in a decrease in the median values and diagnostic sensitivity of the aforementioned biomarkers (serum ESR, CRP, synovial WBC, and PMN%), but this was not observed for leukocyte esterase [70]. In addition to the practical benefits of being point-of-care tests with immediate results, these findings demonstrate that synovial fluid alpha-defensin assays and leukocyte esterase strip tests maintain their diagnostic accuracy even in the unfortunate but not uncommon circumstance wherein antibiotics have been administered prematurely, unlike serum ESR and CRP as well as synovial fluid WBC count and PMN%. For cases in which antibiotics have been administered prior to diagnostic workup, current evidence suggests that synovial alpha-defensin assays and leukocyte esterase strip tests may be used as reliable tools to support or reject the diagnosis of PJI in conjunction with the clinical presentation and other diagnostic criteria. 

## 5. Current Solutions: Sonication of Implants

It is possible that newer techniques for microbiological identification such as sonication fluid cultures could prove to be useful in these circumstances. Current evidence suggests that low-intensity sonication of explanted prostheses is an effective means to disrupt biofilm on the prosthetic surface to increase the sensitivity of microbiological isolation compared to traditional sampling of synovial fluid or periprosthetic tissues [37,71,72]. Sonication may also improve culture yield by dislodging sessile organisms on explanted prostheses [73]. Cultures of sonication fluid have demonstrated an improved sensitivity (78–97%) in microorganism identification without compromising specificity (81–99%) [37,74,75,76]. In a key study by Trampuz et al., the authors found a sensitivity of 79% for sonication fluid cultures, which was significantly greater than that of tissue cultures (61%) [37]. More importantly, these findings persisted even in the presence of antimicrobial therapy within 14 days prior to surgery (75% vs. 45%). The superior diagnostic accuracy of sonication fluid for microorganism identification was confirmed in a recent meta-analysis of 12 studies, which reported a pooled sensitivity and specificity of 80% (95% CI, 0.74 to 0.84) and 95% (95% CI, 0.90 to 0.98), respectively [77]. Despite these promising results, some authors have suggested that the accuracy of sonication fluid cultures may vary based on the sonication technique used [78] as well as timing of PJI [79]. False-positive results have also been observed and attributed to contamination during the sonication process [80]. To overcome this limitation, most authors have recommended a diagnostic threshold of at least five colony-forming units (CFUs) for sonication fluid cultures [37,76,77]. In view of the overwhelming evidence demonstrating improved pathogen isolation with the use of sonication fluid cultures relative to traditional synovial fluid or tissue cultures, sonication of explanted prostheses for microbiological identification could be particularly useful in the context of premature antibiotic administration wherein the risk of CN-PJI is far greater.

## 6. Future Solutions: Molecular Testing

The reliance on culture as the gold standard for diagnosis has led to the conundrum of CN-PJI. Molecular techniques to detect bacterial DNA present a unique opportunity to improve the accuracy of diagnosis for PJI, particularly in the setting of negative cultures [12]. Multiplex polymerase chain reaction (PCR)-based assays allow the detection of common microorganisms and their resistance genes, improving sensitivity and reducing the time to diagnosis compared with traditional cultures [81,82]. However, the requirement for specific primers often results in the failure to detect atypical or less common pathogens as well as resistance mechanisms [83]. Another molecular technique currently available is 16S rRNA gene sequencing [81]. Unlike PCR-based assays, this method allows the detection of a wider variety of bacterial species, prompting some authors to suggest that 16S rRNA sequencing may have a higher sensitivity compared to bacterial cultures and PCR-based techniques [81,84,85]. Primers used in this technique are specific for highly conserved sequences that are found in almost all bacteria, as well as variable regions in between them, thereby allowing the identification of a broad range of bacteria. However, major limitations of this method include the inability to detect antimicrobial resistance genes and polymicrobial infections, which can only be determined using high-throughput sequencing methods rather than traditional capillary-based ones. More recently, metagenomic next generation sequencing (mNGS) has been developed to overcome the shortcomings of previous molecular tests. This high-throughput sequencing technique enables the detection of complete bacterial genomes, including unculturable, unsuspected, and non-viable organisms in the sample [86,87,88,89]. Resistance genes can also be simultaneously detected using this technique [88]. Direct sequencing of specimens improves the diagnostic yield compared to traditional cultures [87], as recent studies have shown that mNGS was able to detect new organisms in 16–44% of CN-PJI cases and 4–67% of culture-positive cases [86,87,88,89]. 

More importantly, current evidence suggests that molecular methods for pathogen identification are unaffected by prior antibiotic administration. Fang et al. studied 8 patients who had antibiotic treatment prior to the diagnosis of CN-PJI and found that 3 of 8 had positive rRNA-PCR and 6 of 8 had positive DNA-PCR [90]. In another study of 144 patients with PJI, Cazanave et al. reported that 69.7% of patients receiving antimicrobial therapy within 14 days of surgery had bacteria isolated on tissue and sonicate fluid cultures, whereas 87.9% of patients had bacteria detected from sonicate fluid using PCR [83]. Similarly, a higher proportion of patients on antibiotic treatment within 28 days of surgery had a positive PCR compared to those who had a positive sonicate fluid or tissue culture. These findings led the authors to conclude that PCR assays were less affected by antibiotic administration compared to traditional cultures, highlighting the persistence of microbial DNA in synovial fluid and tissue specimens following a prolonged course of antimicrobial treatment [91]. Overall, molecular techniques show considerable promise for diagnosing PJI in patients who inadvertently received antibiotics before the collection of intraoperative samples, overcoming the limitations of traditional cultures. Another area that these newer techniques can be applied to is the management of patients undergoing two-stage exchange arthroplasty. The first stage of this procedure involves the resection of all components, aggressive debridement, and insertion of an antibiotic-loaded cement spacer. This is followed by an interim stage of prolonged antibiotic administration that is guided by the susceptibility of pathogen(s) isolated from intraoperative cultures. The second and final stage involves the removal of the spacer, repeat debridement, and implantation of new prostheses. As it is often difficult to ascertain whether infection has been eradicated following a course of four to six weeks of systemic antibiotics in the interim stage, current practice often involves rechecking inflammatory markers such as ESR and CRP, although this has been shown to correlate poorly with the likelihood of residual infection at the time of reimplantation [92,93,94]. Alternatively, synovial fluid cultures may be taken after an “antibiotic holiday” of two weeks to improve diagnostic yield, prior to new prosthesis implantation. In such cases, molecular testing not only circumvents the need for an “antibiotic holiday”, but also provides rapidly available, more sensitive diagnostic information that can guide clinical decisions such as the appropriateness and timing of reimplantation [95]. The utility of molecular methods may also extend to patients on chronic suppressive antibiotic therapy, providing a reliable method for monitoring bacterial load as well as the development of antimicrobial resistance. However, it is important to note that while the ability to detect bacterial DNA even after cell death from antimicrobial therapy may seem advantageous in these situations, this is in fact a double-edged sword as these techniques cannot differentiate between active versus eradicated infections [91], and previous studies have demonstrated that DNA can also be isolated from non-viable bacteria in sterile joints, especially in cases of inflammatory arthritis [96]. Consequently, the importance of clinical correlation and adjunctive tests to support the diagnosis of PJI cannot be further emphasized [49]. Currently, high costs and complex laboratory workflows are the main obstacles hindering the adoption of molecular testing. As these methods become more cost-efficient over time, their speed of detection as well as improved sensitivity in the setting of prior antibiotic administration compared to traditional cultures will allow clinicians to initiate targeted antimicrobial therapy at an earlier time, potentially improving the treatment outcomes for PJI in the future. 

## 7. Conclusions

No single test can confirm the diagnosis of PJI, hence current diagnostic criteria are based on both clinical as well as laboratory findings [49]. Despite recent guidelines, it is not uncommon to encounter patients with suspected PJI who have already been prescribed antimicrobial therapy by their primary provider. This confounds the diagnostic picture as it interferes with the identification of the causative organism on routine cultures and affects the accuracy of commonly used biomarkers, resulting in confusion for patients and clinicians regarding the diagnosis as well as the inability to administer culture-directed antimicrobials that could increase the chance of infection eradication. Based on current literature, it is the recommendation of the authors that pre-incision prophylactic antibiotics should not be withheld for cases of suspected PJI, but therapeutic antibiotics for the treatment of the infected joint or any concurrent infection (e.g., urinary tract infection) should be withheld for at least two weeks prior to the collection of intraoperative cultures for otherwise medically-stable patients, since the early initiation of antimicrobial therapy is unlikely to be associated with improved chances of treatment success. Exceptions to this rule include patients in whom the causative organism has been reliably identified prior to surgical treatment (e.g., on preoperative joint aspiration). Another exception pertains to a patient with suspected PJI due to bacteremia from another source (e.g., bacterial endocarditis). Such cases should be managed according to clinical judgement—as the need to treat the cardiac source of infection takes precedence, the patient should be promptly started on intravenous antibiotics. However, if the patient is medically stable, blood cultures should be taken prior to antibiotics, as this would not only guide antibiotic selection when treating the cardiac source, but also possibly isolate the causative organism for the infected joint. Similarly, for unstable patients in whom the infected joint has been determined to be the source of sepsis, early collection of cultures (e.g., joint aspiration and/or blood cultures) and initiation of antibiotic therapy should be prioritized. In the event that a patient inadvertently received antibiotics prior to the collection of cultures, newer molecular techniques could be used to identify the infecting organism, especially in culture-negative cases, since their diagnostic accuracy is maintained even in patients who receive antimicrobials prematurely. It is possible that once these highly sensitive molecular methods gain widespread adoption, clinicians will be prompted to question the necessity of withholding antibiotics until intraoperative sampling is performed. Until then, it is imperative that primary care and emergency providers recognize the implications of premature antibiotic administration as outlined in this article, as this can render an already challenging orthopedic complication even more difficult to manage and increase morbidity risk for the patient. 

## Data Availability

Data sharing not applicable.

## References

[1] Namba R.S., Inacio M.C., Paxton E.W. (2013). Risk factors associated with deep surgical site infections after primary total knee arthroplasty: An analysis of 56,216 knees. JBJS.

[2] Kurtz S.M., Ong K.L., Lau E., Bozic K.J., Berry D., Parvizi J. (2010). Prosthetic joint infection risk after TKA in the Medicare population. Clin. Orthop. Relat. Res..

[3] Ong K.L., Kurtz S.M., Lau E., Bozic K.J., Berry D.J., Parvizi J. (2009). Prosthetic joint infection risk after total hip arthroplasty in the Medicare population. J. Arthroplast..

[4] Sloan M., Premkumar A., Sheth N.P. (2018). Projected Volume of Primary Total Joint Arthroplasty in the U.S., 2014 to 2030. JBJS.

[5] Premkumar A., Kolin D.A., Farley K.X., Wilson J.M., McLawhorn A.S., Cross M.B., Sculco P.K. (2020). Projected Economic Burden of Periprosthetic Joint Infection of the Hip and Knee in the United States. J. Arthroplast..

[6] Parvizi J., Fassihi S.C., Enayatollahi M.A. (2016). Diagnosis of Periprosthetic Joint Infection Following Hip and Knee Arthroplasty. Orthop. Clin. N. Am..

[7] Yang J., Parvizi J., Hansen E.N., Culvern C.N., Segreti J.C., Tan T., Hartman C.W., Sporer S.M., Della Valle C.J. (2020). The Knee Society Research Group 2020 Mark Coventry Award: Microorganism-directed oral antibiotics reduce the rate of failure due to further infection after two-stage revision hip or knee arthroplasty for chronic infection: A multicentre randomized controlled trial at a minimum of two years. Bone Jt. J..

[8] Kalbian I., Park J.W., Goswami K., Lee Y.-K., Parvizi J., Koo K.-H. (2020). Culture-negative periprosthetic joint infection: Prevalence, aetiology, evaluation, recommendations, and treatment. Int. Orthop..

[9] McNally M., Sousa R., Wouthuyzen-Bakker M., Chen A.F., Soriano A., Vogely H.C., Clauss M., Higuera C.A., Trebše R. (2021). The EBJIS definition of periprosthetic joint infection. Bone Jt. J..

[10] Ascione T., Barrack R., Benito N., Blevins K., Brause B., Cornu O., Frommelt L., Gant V., Goswami K., Hu R. (2019). General Assembly, Diagnosis, Pathogen Isolation—Culture Matters: Proceedings of International Consensus on Orthopedic Infections. J. Arthroplast..

[11] Berbari E., Marculescu C., Sia I., Lahr B.D., Hanssen A.D., Steckelberg J.M., Gullerud R., Osmon D.R. (2007). Culture-Negative Prosthetic Joint Infection. Clin. Infect. Dis..

[12] Parvizi J., Erkocak O.F., Della Valle C.J. (2014). Culture-Negative Periprosthetic Joint Infection. JBJS.

[13] American Academy of Orthopaedic Surgeons American Academy of Orthopaedic Surgeons Clinical Practice Guideline on the Diagnosis and Prevention of Periprosthetic Joint Infections. AAOS Quality & Practice Resources n.d. https://www.aaos.org/contentassets/9a006edd608c468ba066624defca5502/pji-clinical-practice-guideline-final-9-18-19-.pdf.

[14] Huotari K., Peltola M., Jämsen E. (2015). The incidence of late prosthetic joint infections: A registry-based study of 112,708 primary hip and knee replacements. Acta Orthop..

[15] Zeller V., Kerroumi Y., Meyssonnier V., Heym B., Metten M.-A., Desplaces N., Marmor S. (2018). Analysis of postoperative and hematogenous prosthetic joint-infection microbiological patterns in a large cohort. J. Infect..

[16] Naber C.K. (2009). *Staphylococcus aureus* Bacteremia: Epidemiology, Pathophysiology, and Management Strategies. Clin. Infect. Dis..

[17] Sendi P., Banderet F., Graber P., Zimmerli W. (2011). Clinical comparison between exogenous and haematogenous periprosthetic joint infections caused by *Staphylococcus aureus*. Clin. Microbiol. Infect..

[18] Rakow A., Perka C., Trampuz A., Renz N. (2019). Origin and characteristics of haematogenous periprosthetic joint infection. Clin. Microbiol. Infect..

[19] Maderazo E.G., Judson S., Pasternak H. (1988). Late infections of total joint prostheses. A review and recommendations for prevention. Clin. Orthop. Relat. Res..

[20] Tande A.J., Patel R. (2014). Prosthetic Joint Infection. Clin. Microbiol. Rev..

[21] Karim M.A., Andrawis J., Bengoa F., Bracho C., Compagnoni R., Cross M., Danoff J., Della Valle C.J., Foguet P., Fraguas T. (2019). Hip and Knee Section, Diagnosis, Algorithm: Proceedings of International Consensus on Orthopedic Infections. J. Arthroplast..

[22] Hughes J.G., Vetter E.A., Patel R., Schleck C.D., Harmsen S., Turgeant L.T., Franklin R. (2001). Culture with BACTEC Peds Plus/F bottle compared with conventional methods for detection of bacteria in synovial fluid. J. Clin. Microbiol..

[23] Malekzadeh D., Osmon D.R., Lahr B.D., Hanssen A.D., Berbari E.F. (2010). Prior Use of Antimicrobial Therapy is a Risk Factor for Culture-negative Prosthetic Joint Infection. Clin. Orthop. Relat. Res..

[24] Pasquaroli S., Zandri G., Vignaroli C., Vuotto C., Donelli G., Biavasco F. (2013). Antibiotic pressure can induce the viable but non-culturable state in *Staphylococcus aureus* growing in biofilms. J. Antimicrob. Chemother..

[25] Pasquaroli S., Citterio B., Di Cesare A., Amiri M., Manti A., Vuotto C., Biavasco F. (2014). Role of Daptomycin in the Induction and Persistence of the Viable but Non-Culturable State of *Staphylococcus aureus* Biofilms. Pathogens.

[26] Zhao X., Zhong J., Wei C., Lin C.-W., Ding T. (2017). Current Perspectives on Viable but Non-culturable State in Foodborne Pathogens. Front. Microbiol..

[27] Li L., Mendis N., Trigui H., Oliver J.D., Faucher S.P. (2014). The importance of the viable but non-culturable state in human bacterial pathogens. Front. Microbiol..

[28] Oliver J.D. (2010). Recent findings on the viable but nonculturable state in pathogenic bacteria. FEMS Microbiol. Rev..

[29] Dworkin J., Shah I.M. (2010). Exit from dormancy in microbial organisms. Nat. Rev. Genet..

[30] Oliver J.D. (2005). The Viable but Nonculturable State in Bacteria. J. Microbiol..

[31] del Mar Lleò M., Benedetti D., Tafi M.C., Signoretto C., Canepari P. (2007). Inhibition of the resuscitation from the viable but non-culturable state in Enterococcus faecalis. Environ. Microbiol..

[32] Tan T.L., Kheir M.M., Shohat N., Tan D.D., Kheir M., Chen C., Parvizi J. (2018). Culture-Negative Periprosthetic Joint Infection: An Update on What to Expect. JBJS Open Access.

[33] Scheer C.S., Fuchs C., Gründling M., Vollmer M., Bast J., Bohnert J.A., Zimmermann K., Hahnenkamp K., Rehberg S., Kuhn S.-O. (2019). Impact of antibiotic administration on blood culture positivity at the beginning of sepsis: A prospective clinical cohort study. Clin. Microbiol. Infect..

[34] Cascone V., Cohen R.S., Dodson N.P., Cannon C.M. (2019). Implications of culture collection after the first antimicrobial dose in septic emergency department patients. Am. J. Emerg. Med..

[35] Cheng M.P., Stenstrom R., Paquette K., Stabler S.N., Akhter M., Davidson A.C., Gavric M., Lawandi A., Jinah R., Saeed Z. (2019). Blood Culture Results Before and After Antimicrobial Administration in Patients With Severe Manifestations of Sepsis: A Diagnostic Study. Ann. Intern. Med..

[36] Geer J.H., Siegel M.D. (2019). Antibiotics and the Yield of Blood Cultures: Sequence Matters. Ann. Intern. Med..

[37] Trampuz A., Piper K.E., Jacobson M.J., Hanssen A.D., Unni K.K., Osmon D.R., Mandrekar J.N., Cockerill F.R., Steckelberg J.M., Greenleaf J.F. (2007). Sonication of Removed Hip and Knee Prostheses for Diagnosis of Infection. N. Engl. J. Med..

[38] Shahi A., Deirmengian C., Higuera C., Chen A., Restrepo C., Zmistowski B., Parvizi J. (2015). Premature Therapeutic Antimicrobial Treatments Can Compromise the Diagnosis of Late Periprosthetic Joint Infection. Clin. Orthop. Relat. Res..

[39] Osmon D.R., Berbari E.F., Berendt A.R., Lew D., Zimmerli W., Steckelberg J.M., Rao N., Hanssen A., Wilson W.R. (2013). Diagnosis and Management of Prosthetic Joint Infection: Clinical Practice Guidelines by the Infectious Diseases Society of America. Clin. Infect. Dis..

[40] Barrack R.L., Jennings R.W., Wolfe M.W., Bertot A.J. (1997). The Value of Preoperative Aspiration before Total Knee Revision. Clin. Orthop. Relat. Res..

[41] Mont M.A., Waldman B.J., Hungerford D.S. (2000). Evaluation of Preoperative Cultures before Second-Stage Reimplantation of a Total Knee Prosthesis Complicated by Infection. JBJS.

[42] Tan T.L., Shohat N., Rondon A.J., Foltz C., Goswami K., Ryan S.P., Seyler T.M., Parvizi J. (2019). Perioperative Antibiotic Prophylaxis in Total Joint Arthroplasty: A Single Dose Is as Effective as Multiple Doses. JBJS.

[43] Pérez-Prieto D., Portillo M.E., Puig L., Alier A., Gamba C., Guirro P., Martínez-Díaz S., Horcajada J.P., Trampuz A., Monllau J.C. (2016). Preoperative antibiotic prophylaxis in prosthetic joint infections: Not a concern for intraoperative cultures. Diagn. Microbiol. Infect. Dis..

[44] Tetreault M.W., Wetters N.G., Aggarwal V., Mont M., Parvizi J., Della Valle C.J. (2014). The Chitranjan Ranawat Award: Should Prophylactic Antibiotics Be Withheld Before Revision Surgery to Obtain Appropriate Cultures?. Clin. Orthop. Relat. Res..

[45] Bedenčič K., Kavčič M., Faganeli N., Mihalič R., Mavčič B., Dolenc J., Bajc Z., Trebše R. (2016). Does Preoperative Antimicrobial Prophylaxis Influence the Diagnostic Potential of Periprosthetic Tissues in Hip or Knee Infections?. Clin. Orthop. Relat. Res..

[46] Wouthuyzen-Bakker M., Benito N., Soriano A. (2017). The Effect of Preoperative Antimicrobial Prophylaxis on Intraoperative Culture Results in Patients with a Suspected or Confirmed Prosthetic Joint Infection: A Systematic Review. J. Clin. Microbiol..

[47] Wouthuyzen-Bakker M., Tornero E., Claret G., Bosch J., Martinez-Pastor J.C., Combalia A., Soriano A. (2017). Withholding Preoperative Antibiotic Prophylaxis in Knee Prosthesis Revision: A Retrospective Analysis on Culture Results and Risk of Infection. J. Arthroplast..

[48] Spangehl M.J., Masri B.A., O’connell J.X., Duncan C.P. (1999). Prospective Analysis of Preoperative and Intraoperative Investigations for the Diagnosis of Infection at the Sites of Two Hundred and Two Revision Total Hip Arthroplasties. JBJS.

[49] Parvizi J., Tan T.L., Goswami K., Higuera C., Della Valle C., Chen A.F., Shohat N. (2018). The 2018 Definition of Periprosthetic Hip and Knee Infection: An Evidence-Based and Validated Criteria. J. Arthroplast..

[50] Nora D., Salluh J., Martin-Loeches I., Póvoa P. (2017). Biomarker-guided antibiotic therapy—strengths and limitations. Ann. Transl. Med..

[51] Rubin B.K., Tamaoki J. (2005). Antibiotics as Anti-Inflammatory and Immunomodulatory Agents.

[52] Iino Y., Toriyama M., Natori Y., Kudo K., Yuo A. (1992). Erythromycin inhibition of lipopolysaccharide-stimulated tumor necrosis factor alpha production by human monocytes in vitro. Ann. Otol. Rhinol. Laryngol..

[53] Bailly S., Fay M., Ferrua B., Gougerot-Pocidalo M.A. (2008). Ciprofloxacin treatment in vivo increases the ex vivo capacity of lipopolysaccharide-stimulated human monocytes to produce IL-1, IL-6 and tumour necrosis factor-alpha. Clin. Exp. Immunol..

[54] Baumann H., Gauldie J. (1994). The acute phase response. Immunol. Today.

[55] Trampuz A., Hanssen A.D., Osmon D.R., Mandrekar J., Steckelberg J.M., Patel R. (2004). Synovial fluid leukocyte count and differential for the diagnosis of prosthetic knee infection. Am. J. Med..

[56] Zmistowski B., Restrepo C., Huang R., Hozack W.J., Parvizi J. (2012). Periprosthetic Joint Infection Diagnosis. J. Arthroplast..

[57] Xie K., Dai K., Qu X., Yan M. (2017). Serum and Synovial Fluid Interleukin-6 for the Diagnosis of Periprosthetic Joint Infection. Sci. Rep..

[58] Berbari E., Mabry T., Tsaras G., Spangehl M., Erwin P.J., Murad M.H., Steckelberg J., Osmon D. (2010). Inflammatory Blood Laboratory Levels as Markers of Prosthetic Joint Infection: A Systematic Review and Meta-Analysis. JBJS.

[59] Ettinger M., Calliess T., Kielstein J.T., Sibai J., Brückner T., Lichtinghagen R., HenningWindhagen H., Lukasz A. (2015). Circulating Biomarkers for Discrimination Between Aseptic Joint Failure, Low-Grade Infection, and High-Grade Septic Failure. Clin. Infect. Dis..

[60] Parvizi J., Jacovides C., Antoci V., Ghanem E. (2011). Diagnosis of periprosthetic joint infection: The utility of a simple yet unappreciated enzyme. JBJS.

[61] Tischler E.H., Cavanaugh P.K., Parvizi J. (2014). Leukocyte Esterase Strip Test: Matched for Musculoskeletal Infection Society Criteria. JBJS.

[62] Wetters N., Berend K.R., Lombardi A.V., Morris M.J., Tucker T.L., Della Valle C.J. (2012). Leukocyte Esterase Reagent Strips for the Rapid Diagnosis of Periprosthetic Joint Infection. J. Arthroplast..

[63] Deirmengian C., Kardos K., Kilmartin P., Gulati S., Citrano P., Booth R.E. (2015). The Alpha-defensin Test for Periprosthetic Joint Infection Responds to a Wide Spectrum of Organisms. Clin. Orthop. Relat. Res..

[64] Mathew B., Nagaraj R. (2015). Antimicrobial activity of human α-defensin 5 and its linear analogs: N-terminal fatty acylation results in enhanced antimicrobial activity of the linear analogs. Peptides.

[65] Xie Z., Feng J., Yang W., Xiang F., Yang F., Zhao Y., Cao Z., Li W., Chen Z., Wu Y. (2015). Human α-defensins are immune-related Kv1.3 channel inhibitors: New support for their roles in adaptive immunity. FASEB J..

[66] Bingham J., Clarke H.D., Spangehl M.J., Schwartz A.J., Beauchamp C.P., Goldberg B. (2014). The Alpha Defensin-1 Biomarker Assay can be Used to Evaluate the Potentially Infected Total Joint Arthroplasty. Clin. Orthop. Relat. Res..

[67] Frangiamore S.J., Gajewski N.D., Saleh A., Kovac M.F., Barsoum W.K., Higuera C.A. (2016). α-Defensin Accuracy to Diagnose Periprosthetic Joint Infection—Best Available Test?. J. Arthroplast..

[68] Deirmengian C., Kardos K., Kilmartin P., Cameron A., Schiller K., Parvizi J. (2014). Combined Measurement of Synovial Fluid α-Defensin and C-Reactive Protein Levels: Highly Accurate for Diagnosing Periprosthetic Joint Infection. JBJS.

[69] Shahi A., Parvizi J., Kazarian G.S., Higuera C., Frangiamore S., Bingham J., Beauchamp C., Della Valle C., Deirmengian C. (2016). The Alpha-defensin Test for Periprosthetic Joint Infections Is Not Affected by Prior Antibiotic Administration. Clin. Orthop. Relat. Res..

[70] Shahi A., Alvand A., Ghanem E., Restrepo C., Parvizi J. (2019). The Leukocyte Esterase Test for Periprosthetic Joint Infection Is Not Affected by Prior Antibiotic Administration. JBJS.

[71] Shen H., Tang J., Wang Q., Jiang Y., Zhang X. (2014). Sonication of Explanted Prosthesis Combined with Incubation in BD Bactec Bottles for Pathogen-Based Diagnosis of Prosthetic Joint Infection. J. Clin. Microbiol..

[72] Hischebeth G.T., Randau T.M., Molitor E., Wimmer M.D., Hoerauf A., Bekeredjian-Ding I., Gravius S. (2016). Comparison of bacterial growth in sonication fluid cultures with periprosthetic membranes and with cultures of biopsies for diagnosing periprosthetic joint infection. Diagn. Microbiol. Infect. Dis..

[73] Scorzolini L., Lichtner M., Iannetta M., Mengoni F., Russo G., Panni A.S., Vasto M., Bove M., Villani C., Mastroianni C.M. (2014). Sonication technique im-proves microbiological diagnosis in patients treated with antibiotics before surgery for prosthetic joint infections. New Microbiol..

[74] Puig-Verdié L., Alentorn-Geli E., González-Cuevas A., Sorlí L., Salvadó M., Alier A., Pelfort X., Portillo M.E., Horcajada J.P. (2013). Implant sonication increases the diagnostic accuracy of infection in patients with delayed, but not early, orthopaedic implant failure. Bone Jt. J..

[75] Janz V., Wassilew G.I., Hasart O., Matziolis G., Tohtz S., Perka C. (2013). Evaluation of sonicate fluid cultures in comparison to histological analysis of the periprosthetic membrane for the detection of periprosthetic joint infection. Int. Orthop..

[76] Rothenberg A.C., Wilson A.E., Hayes J.P., O’Malley M.J., Klatt B.A. (2017). Sonication of Arthroplasty Implants Improves Accuracy of Periprosthetic Joint Infection Cultures. Clin. Orthop. Relat. Res..

[77] Zhai Z., Li H., Qin A., Liu G., Liu X., Wu C., Zhu Z., Qu X., Dai K., Li H. (2014). Meta-Analysis of Sonication Fluid Samples from Prosthetic Components for Diagnosis of Infection after Total Joint Arthroplasty. J. Clin. Microbiol..

[78] Van Diek F.M., Albers C.G.M., Van Hooff M.L., Meis J.F., Goosen J.H.M. (2017). Low sensitivity of implant sonication when screening for infection in revision surgery. Acta Orthop..

[79] Prieto-Borja L., Auñón Á., Blanco A., Fernández-Roblas R., Gadea I., García-Cañete J., Parrón R., Esteban J. (2018). Evaluation of the use of sonication of retrieved implants for the diagnosis of prosthetic joint infection in a routine setting. Eur. J. Clin. Microbiol. Infect. Dis..

[80] Janz V., Wassilew G.I., Hasart O., Tohtz S., Perka C. (2013). Improvement in the detection rate of PJI in total hip arthroplasty through multiple sonicate fluid cultures: Multiple Sonicate Cultures for PJI. J. Orthop. Res..

[81] Janz V., Schoon J., Morgenstern C., Preininger B., Reinke S., Duda G., Breitbach A., Perka C.F., Geissler S. (2018). Rapid detection of periprosthetic joint infection using a combination of 16s rDNA polymerase chain reaction and lateral flow immunoassay. Bone Jt. Res..

[82] Sigmund I.K., Holinka J., Sevelda F., Staats K., Heisinger S., Kubista B., McNally M.A., Windhager R. (2019). Performance of automated multiplex polymerase chain reaction (mPCR) using synovial fluid in the diagnosis of native joint septic arthritis in adults. Bone Jt. J..

[83] Cazanave C., Greenwood-Quaintance K.E., Hanssen A.D., Karau M.J., Schmidt S.M., Urena E.O.G., Mandrekar J.N., Osmon D.R., Lough L.E., Pritt B.S. (2013). Rapid Molecular Microbiologic Diagnosis of Prosthetic Joint Infection. J. Clin. Microbiol..

[84] Tsang S.-T.J., McHugh M.P., Guerendiain D., Gwynne P.J., Boyd J., Simpson A.H.R.W., Walsh T.S., Laurenson I.F., Templeton K.E. (2018). Underestimation of *Staphylococcus aureus* (MRSA and MSSA) carriage associated with standard culturing techniques. Bone Jt. Res..

[85] Chen M.-F., Chang C.-H., Chiang-Ni C., Hsieh P.-H., Shih H.-N., Ueng S.W.N., Chang Y. (2019). Rapid analysis of bacterial composition in prosthetic joint infection by 16S rRNA metagenomic sequencing. Bone Jt. Res..

[86] Tarabichi M., Alvand A., Shohat N., Goswami K., Parvizi J. (2018). Diagnosis of Streptococcus canis periprosthetic joint infection: The utility of next-generation sequencing. Arthroplast. Today.

[87] Street T.L., Sanderson N.D., Atkins B.L., Brent A.J., Cole K., Foster D., . McNally M.A., Oakley S., Peto L., Taylor A. (2017). Molecular diagnosis of or-thopaedic device infection direct from sonication fluid by metagenomic sequencing. bioRxiv.

[88] Ruppé E., Lazarevic V., Girard M., Mouton W., Ferry T., Laurent F., Schrenzel J. (2017). Clinical metagenomics of bone and joint infections: A proof of concept study. Sci. Rep..

[89] Thoendel M.J., Jeraldo P.R., Greenwood-Quaintance K.E., Yao J., Chia N., Hanssen A.D., Abdel M.P., Patel R. (2018). Identification of Prosthetic Joint Infection Pathogens Using a Shotgun Metagenomics Approach. Clin. Infect. Dis..

[90] Fang X.-Y., Li W.-B., Zhang C.-F., Huang Z.-D., Zeng H.-Y., Dong Z., Zhang W.-M. (2018). Detecting the Presence of Bacterial DNA and RNA by Polymerase Chain Reaction to Diagnose Suspected Periprosthetic Joint Infection after Antibiotic Therapy. Orthop. Surg..

[91] Van Der Heijden I.M., Wilbrink B., Vije A.E., Schouls L.M., Breedveld F.C., Tak P.P. (1999). Detection of bacteri-al DNA in serial synovial samples obtained during antibiotic treatment from patients with septic ar-thritis. Arthritis Rheum. Off. J. Am. Coll. Rheumatol..

[92] Kusuma S.K., Ward J., Jacofsky M., Sporer S.M., Della Valle C.J. (2011). What is the Role of Serological Testing Between Stages of Two-stage Reconstruction of the Infected Prosthetic Knee?. Clin. Orthop. Relat. Res..

[93] Shukla S.K., Ward J.P., Jacofsky M.C., Sporer S.M., Paprosky W.G., Della Valle C.J. (2010). Perioperative Testing for Persistent Sepsis Following Resection Arthroplasty of the Hip for Periprosthetic Infection. J. Arthroplast..

[94] Melendez D.P., Greenwood-Quaintance K.E., Berbari E.F., Osmon D.R., Mandrekar J.N., Hanssen A.D., Patel R. (2015). Evaluation of a Genus- and Group-Specific Rapid PCR Assay Panel on Synovial Fluid for Diagnosis of Prosthetic Knee Infection. J. Clin. Microbiol..

[95] Tan T.L., Gomez M.M., Manrique J., Parvizi J., Chen A.F. (2016). Positive Culture During Reimplantation Increases the Risk of Subsequent Failure in Two-Stage Exchange Arthroplasty. JBJS.

[96] Chen T., Rimpiläinen M., Luukkainen R., Möttönen T.T., Yli-Jama T., Jalava J., Vainio O., Toivanen P. (2003). Bacterial components in the synovial tissue of patients with advanced rheumatoid arthritis or osteoarthritis: Analysis with gas chromatography-mass spectrometry and pan-bacterial polymerase chain reaction. Arthritis Rheum..

